# Isoquinoline Alkaloids Isolated from *Corydalis yanhusuo* and Their Binding Affinities at the Dopamine D_1_ Receptor

**DOI:** 10.3390/molecules13092303

**Published:** 2008-09-25

**Authors:** Zhong-Ze Ma, Wei Xu, Niels H. Jensen, Bryan L. Roth, Lee-Yuan Liu-Chen, David Y. W. Lee

**Affiliations:** 1 Bio-Organic and Natural Products Laboratory, McLean Hospital, Harvard Medical School, 115 Mill Street, Belmont, MA 02478, USA; 2 Department of Pharmacology and Center for Substance Abuse Research, School of Medicine, Temple University, 3420 N. Broad St, Philadelphia, PA 19140, USA; E-mail: wxu00001@temple.edu (W. X.), lliuche@temple.edu (L-Y. L-C.); 3 Department of Pharmacology, University of North Carolina Medical School, Chapel Hill, NC 27599, USA; E-mail: niels_h_jensen@hotmail.com (N-H. J.); 4 Center for Neurobiology Division of Medicinal Chemistry and Natural Products, and NIMH Psychoactive Drug Screening Program, University of North Carolina Medical School, Chapel Hill, NC 27599, USA; E-mail: bryan_roth@med.unc.edu (B-L. R.)

**Keywords:** *Corydalis yanhusuo*, Isoquinoline alkaloids, *d/l* Ratio, Chiral HPLC, Dopamine receptor

## Abstract

Bioactivity-guided fractionation of *Corydalis yanhusuo* has resulted in the isolation of eight known isoquinoline alkaloids - tetrahydropalmatine, isocorypalmine, stylopine, corydaline, columbamine, coptisin, 13-methylpalmatine, and dehydro-corybulbine. The tertiary alkaloids were further analyzed by chiral HPLC to determine the ratios of *d*-and *l*-isomers. The isolated compounds were screened for their binding affinities at the dopamine D_1_ receptor. Isocorypalmine had the highest affinity (*K_i_* = 83 nM). The structure-affinity relationships of these alkaloids are discussed.

## Introduction

Alcohol and drug abuse are major social and medical problems that impose a significant burden on society [1,2]. Because of the complexity of drug dependence and the lack of effective remedy, especially for relapse, the development of pharmacotherapy for its treatment has been a serious challenge [3]. Despite a great deal of effort in developing effective therapies, only few medications have been approved by the U.S. Food and Drug Administration [2, 4]. These medications have shown only limited efficacy, serious side effects and poor compliance in patients [5,6,7,8], therefore, an alternative approach, the systematic evaluation of traditional herbal medicines, may provide new drug candidates for the treatment of alcohol and drug abuse. Chinese herbal medicines have been used historically in the treatment of alcohol and drug abuse [4, 9]. Many are still prescribed in China and Southeast Asia for this purpose, and their clinical efficacy of some medications has recently been documented [10]. NPI-025 [11] is a herbal formula used clinically to treat opioid addiction in Hong Kong. It is composed of five Chinese herbs, including Rhizoma Corydalis (Yan Hu Suo), Rhizoma Et Radix Notopterygh (Qiang Huo), Ramulus Uncariae Cum Uncis (Gou Teng), Rhizoma Chuanxiong (Chuan Xiong) and Radix Aconiti Lateralis Preparata (Fu Zi, treated with hot strong base to reduce toxicity) [12,13].

The dried tuber of *Corydalis yanhusuo* (Papaveraceae) is one of key ingredients in NPI-025. *C. yanhusuo* has been used traditionally to promote blood circulation, reinforce vital energy, and alleviate pain such as headache, chest pain, epigastric pain, abdominal pain, backache, arthralgia, dysmenorrheal pain, or trauma, and is officially listed in the Chinese Pharmacopoeia [14,15,16]. Previous phytochemical and pharmacological studies have identified several alkaloids as the active secondary metabolites of the plant [16]. *dl*-Tetrahydropalmatine (*dl*-THP), one of the major active alkaloids, has been found to be a neuroactive alkaloid [17,18,19]. It has been listed in the Chinese Pharmacopoeia since 1977 as an analgesic with sedative and hypnotic effects. Recent studies have demonstrated that *l*-THP inhibits opiate tolerance and withdrawal syndromes in rats [20,21]. It was also reported that *l*-THP significantly inhibits cocaine- or methamphetamine-induced conditioned place preference [22,23]. In addition, *l*-THP inhibited cocaine-triggered reinstatement [24] and the rewarding effects of cocaine in rats as measured by cocaine self-administration [24,25] and intracranial self-stimulation [25]. In a recent double-blind clinical trial in China, Yang *et al.* [10] found that treatment of 119 heroin-only dependent in-patients with *l*-THP (60 mg orally twice a day) for one month significantly reduced heroin craving and withdrawal symptoms. These findings suggest that *l*-THP could be an excellent drug candidate for treatment of drug addition.

As part of our ongoing investigation of alternative therapies for substance addiction [11, 26], we initiated a comprehensive fractionation study guided by dopamine receptor binding assays in an attempt to find new dopamine receptor agonists or antagonists from *C. yanhusuo*. Herein we report the isolation of eight isoquinoline alkaloids, their D_1_ receptor binding activities and their stereochemistry by chiral HPLC method.

## Results and Discussion

The 70% aqueous acetone extract of *C. yanhusuo* were subjected to sequential extraction with hexane, ethyl acetate, butanol, methanol and water. In our initial biological study as shown in Table 1, the hexane, ethyl acetate, butanol and methanol extracts at 50 mg/mL showed moderate binding activity at rat dopamine D_1_ receptor (rD_1_R) stably expressed in CHO cells, compared with the positive control SKF82958 [27] (10 μM), while the water extract had no activity.

**Table 1:** Displacement of [^3^H]SCH 23390 binding to membranes of CHO cells stably expressing the rD_1_R.

Sample I.D.	Concentration	Displaced [^3^H]SCH 23390
hexane extract	50 mg/mL	100%
AcOEt extract	50 mg/mL	96%
BuOH extract	50 mg/mL	97%
MeOH extract	50 mg/mL	88%
water extract	50 mg/mL	-2%
SKF82958	10 μM	99%

**Scheme 1:**
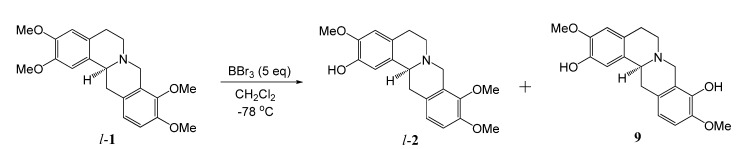
Synthesis of *l*-**2** from *l*-**1.**

**Figure 1:**
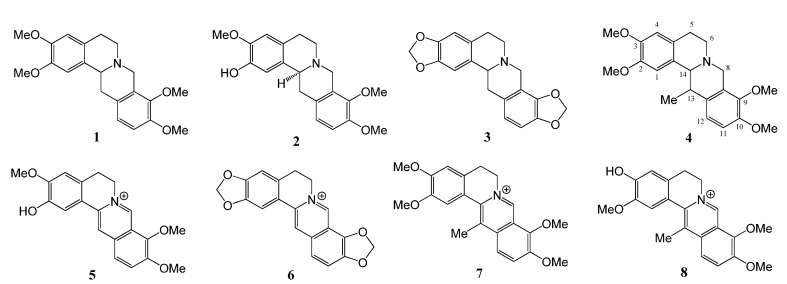
Isoquinoline alkaloids isolated from *C. yanhusuo.*

**Figure 2:**
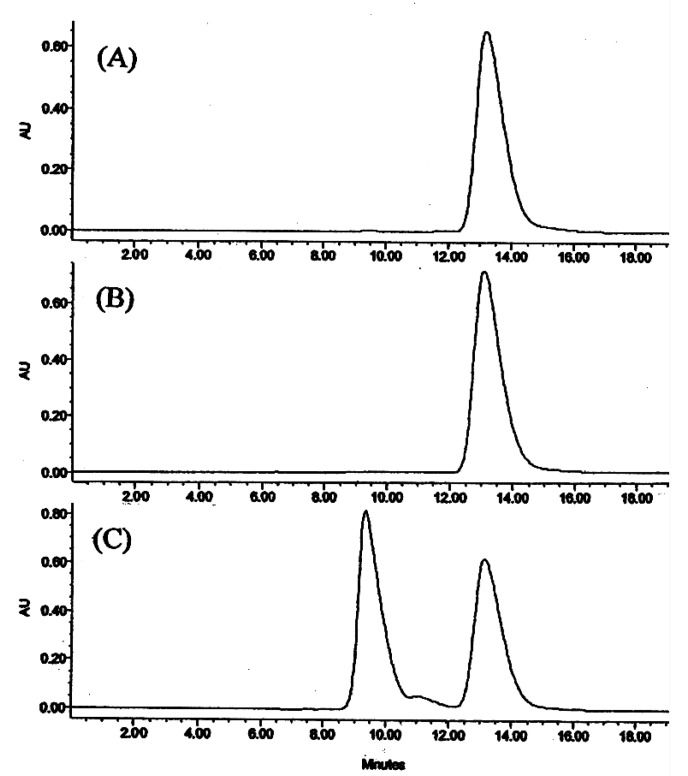
Chromatograms obtained from chiral separation of **2** (A), synthetic *l*-**2** (B) and *dl*-**2** (C).

The hexane, ethyl acetate, butanol and methanol extracts were chromatographed on silica gel columns to give eight known isoquinoline alkaloids (Figure 1), tetrahydropalmatine (**1**) [28], isocorypalmine (**2**) [28], stylopine (**3**) [29], corydaline (**4**) [30], columbamine (**5**) [31], coptisin (**6**) [31], 13-methylpalmatine (**7**) [31] and dehydrocorybulbine (**8**) [32]. The tertiary alkaloids **1**, **2 **and **3** have a chiral center at the C-14 position, and may consist of two optical isomers. Compound **4** has two chiral centers at the C-13 and C-14 positions, and the ^1^H-NMR spectrum of **4** displayed the H-14 methine as a doublet at δ 3.68 (*J* = 2.7 Hz), indicating the protons at H-13 and H-14 are *cis* oriented [30]. Following the reported method [17,18], compounds **1**, **2**, **3** and **4** were analyzed by chiral HPLC. As shown in Table 2, the ratios of *d*- and *l*-isomers of **1**, **3** and **4** are 3:1, 1:10 and 10:1, respectively, while **2** gave only one peak in its chiral HPLC (Figure 2). In order to further confirm the stereochemistry of **2**, *l*-**2** was synthesized by demethylation of *l*-THP (*l*-**1**) with BBr_3_ in CH_2_Cl_2_ (Scheme 1). The reaction time is critical for a good yield. After one hour, the main product was *l*-**2**, while after 2 h the main product was found to be the *l*-scoulerine (**9**). Compounds *l*-**2** and **9** were difficult to be separated by silica gel column chromatography. However, based on its solubility, *l*-**2** can be readily purified by recrystalization from methanol. Following the same synthetic procedure, *dl*-**2** was also prepared from *dl*-THP (*dl*-**1**). Comparison of the chiral HPLC chromatogram of **2** with those of *l*-**2** and *dl*-**2** (Figure 2) confirmed that compound **2** was the *l*-enantiomer.

*HPLC conditions*: Waters 1525 Binary HPLC Pump; Waters 2487 Dual λ Absorbance Detector; Chiralcel OD Column (4.6 x 250 mm); 50% EtOH as mobile phase; Wavelength, 230 nm; Sample concentration, 0.5 mg/mL; Injection volume, 5 µL

The isolated alkaloids were tested for dopamine receptor binding as reported previously [11]. Compounds **1**, **2**, **3**, **5**, **7** and **8** inhibited [^3^H]SCH 23390 binding to human D_1_ receptor (hD_1_R) by more than 50% at 10 μM, and their binding affinities were determined (Table 2). The *l*-enantiomer of **1** (*l*-THP) has a D_1_ affinity of 94 nM while the 3:1 mixture of the *d*- and *l*-enantiomers **1** has a much weaker affinity of 1.3 μM. It has been reported that *l*-**1** has analgesic activity with a more potent tranquilizing effect than the *d* isomer [19, 33]. Previous studies have demonstrated that *l*-**1** acts on dopamine receptors, but *d*-THP does not [34, 35]. Compound *l*-**1** is a non-subtype selective antagonist at dopamine receptors[35,36,37].

Of the isolated alkaloids, compound **2** has the highest D_1_ affinity with a *K*_i_ of 83 nM, comparable in potency to *l*-**1**. Jin et al. [35, 37] reported that *l*-THP derivatives with one hydroxyl group at C-2 and two hydroxyl groups at C-2 and C-9 or C-2 and C-10 were more potent at dopamine receptors than *l*-**1**. On the other hand, Guo et al. [38] reported that *l*-**1** was readily metabolized to desmethylated *l*-THPs in rats.

**Table 2:** Binding affinities of the purified alkaloids for the [^3^H] SCH 23390 labeled the hD_1_R in transiently transfected HEK-T cells.

Compound	ratio of *d*:*l*-isomers	*K_i _*(nM)^a^
**1**	3:1	1,293 ± 71
**2**	0:1	83 ± 5
**3**	1:10	1,043 ± 66
**4**	10:1	>10,000
**5**	-	4,112 ± 274
**6**	-	>10,000
**7**	-	3,867 ± 235
**8**	-	1,836 ± 200
*l*-THP	0:1	94 ± 9

^a ^Each value represents the mean ± SEM of three independent experiments performed in duplicate

As shown in Table 2, the quaternary alkaloids (**5** and **6**) exhibited weaker binding affinities than the corresponding tertiary alkaloids (**2** and **3**). Comparison of the binding affinities of **7** and **8** with that of **5** revealed that the methyl group at C-13 did not affect the overall binding activity at the D_1_ receptor.

In order to obtain enough pure alkaloids for biological and pharmacological studies, another larger batch of crude extract of *C. yanhusuo* was fractionated. Compounds **1**, **2**, **3** and **4** were isolated from the new extract. Subsequent chiral HPLC analysis showed that the ratios of *d*- and *l*-enantiomers in newly isolated **2** and **3** remained the same as those of previous isolated **2** and **3**, while the ratios in both **1** and **4** were changed to 1:1. The changes of these components in different extracts of *C. yanhusuo* indicated that different geographical regions, seasons of harvest, storage, or extraction conditions could affect the chemical composition significantly. As evidenced in our case, quality control of the active optical isomers will be very important for future biological, pharmacological, and clinical studies of *C. yanhusuo*.

## Conclusions

The mesolimbic dopaminergic system has been shown to play an important role in drug abuse [39]. Compounds targeted to these dopamine receptors can provide a rational treatment of drug abuse [40]. The stereochemistry of *l*-isocorypalmine (**2**) and the *d/l* ratio of **1**, **3**, and **4**, were established unambiguously by using a chiral HPLC method along with concise chemical synthesis. In addition, this convenient and effective synthesis for selective cleavage the methoxy group at C-2 position allows the preparation of *l*-isocorypalmine in a large amount from readily available *l*- tetrahydropalmatine for animal study. The present study demonstrates that isoquinoline alkaloids are the principal constituents for the binding activities of *C. yanhusuo* extracts at the dopamine D_1_ receptor. *l*-Isocorypalmine (**2**) showed the highest affinity and is a promising drug candidate. This study provides an important pharmacological basis to support the traditional use of *C. yanhusuo* in the treatment of heroin addiction in China. Its complete pharmacological characterization is under investigation and will be reported in due course.

## Experimental

### General

The NMR spectra were recorded on a Varian VXR300 spectrometer with TMS as the internal standard. EI-MS spectra were obtained on a HP5972 Series Mass spectrometer. HPLC was performed on a Waters (Milford, MA, USA) system comprised of a 1525 Binary HPLC Pump equipped with a Waters 2487 Dual λ Absorbance Detector set at a wavelength of 230 nm, a model 7725i sample injector equipped with a 5 μL loop and a Waters Breeze software package for data collection. Compounds were separated on a Chiralcel OD Column (4.6 x 250 mm). The mobile phase was 50:50 (v/v) ethanol–water. All analysis was performed at a flow-rate of 0.5 mL/min with detection at 230 nm. The mobile phase was filtered through a 0.45 μm filter and degassed. Separations were performed at room temperature. Silica gel (Fisher Scientific, USA) were used for column chromatography. TLC was performed on precoated silica gel 60 F254 plates (Merck, Germany).

### Plant material

The dried tubers of *C. yanhusuo* were collected from Qianxian Town, Dongyang District, Zhejiang province, P.R. China in May, 2005. Its botanical identification was confirmed by Dr. Shilin Chen, the Institute of Medicinal Plant Development (IMPLAD), Beijing, P.R. China. A voucher specimen has been deposited in the IMPLAD, Beijing, P.R. China.

### Extraction and isolation

The dried tubers of *C. yanhusuo* (30 kg) were powdered and extracted with 70% aqueous acetone for three times at room temperature, and the solution was evaporated under reduced pressure to give a residue (2.2 kg). The 70% aqueous acetone extract (200 g) was subjected to sequential extraction with hexane, ethyl acetate, butanol, methanol and water. The combined part (3 g) of the above hexane, ethyl acetate and butanol extracts was chromatographed over silica gel (100 g) using CH_2_Cl_2_ with increasing amounts of MeOH (10:1, 5:1, 3:1, 1:1 and 1:2) to give five fractions A-E. Fraction A was further purified by silica gel column, with a gradient of hexane and acetone to afford **1** (20 mg), **2** (15 mg), **3** (10 mg) and **4** (15 mg). Fraction B was chromatographed over silica gel column with a gradient of CH_2_Cl_2_ and MeOH to afford **5** (10 mg), **7** (250 mg) and **8** (3 mg). Fraction C was purified by silica gel column, with a gradient of CH_2_Cl_2_ and MeOH, to afford **6** (8 mg). In a similar manner, the MeOH extract (8 g) was separated by silica gel column chromatography using CH_2_Cl_2_ with increasing amounts of MeOH to give **5** (20 mg) and **7** (500 mg).

### Synthesis of l-isocorypalmine (**2**)

To a stirred CH_2_Cl_2_ solution (10 mL) of *l*-tetrahydropalmatine (*l*-**1**, 355 mg, 1.0 mmol), BBr_3_ solution (1 mM in CH_2_Cl_2_, 5 mL) was added. The reaction was stirred at -78 ºC for 1 h, and then was warmed to room temperature. The mixture was diluted with CH_2_Cl_2_ (50 mL) and washed with excess saturated NaHCO_3_. The organic extract was dried (MgSO_4_), filtered and concentrated under reduced pressure. The residue was purified by silica gel column chromatography (CH_2_Cl_2_ and MeOH, 20:1), followed by recrystallization in MeOH, to give *l*-**2** (150 mg) and *l*-scoulerine (**9**, 40 mg).

### Competitive inhibition of [^3^H] SCH23390 binding to dopamineD_1_ receptor

Chinese hamster ovary cells (CHO) stably transfected with HA-tagged rat D_1_ dopamine receptor (rD_1_R) were grown in 100-mm culture dishes in Dulbecco's modified Eagle's medium F12 HAM supplemented with 10% fetal calf serum, 0.1 mg/mL hygromycin B, 100 units/mL penicillin and 100 µg/mL streptomycin in a humidified atmosphere consisting of 5% CO_2_ and 95% air at 37°C. Binding to membranes prepared from CHO cells stably transfected with the rD_1_R was conducted with [^3^H]SCH23390 (0.2 nM) in 50mMTris-HCl buffer containing 1 mM EGTA (pH 7.4) (TE buffer) at room temperature for 1 h in duplicate. Nonspecific binding was defined as binding in the presence of Fluphenazine (10 μM). The purified compounds showing more than 50% displacement of [^3^H]SCH23390 at 10 μM were further tested for the affinity (*K_i_*) by using GRAPHPAD PRISM.
